# Vitamin D Supplementation Does Not Impact Resting Metabolic Rate, Body Composition and Strength in Vitamin D Sufficient Physically Active Adults

**DOI:** 10.3390/nu12103111

**Published:** 2020-10-12

**Authors:** Karina Romeu Montenegro, Vinicius Cruzat, Hilton Melder, Angela Jacques, Philip Newsholme, Kagan J. Ducker

**Affiliations:** 1 School of Pharmacy and Biomedical Sciences, Curtin Health Innovation Research Institute, Curtin University, Perth, WA 6102, Australia; Philip.Newsholme@curtin.edu.au; 2 Faculty of Health, Torrens University Australia, Melbourne, VIC 3000, Australia; vinicius.cruzat@laureate.edu.au (V.C.); hiltonmelder@gmail.com (H.M.); 3 School of Physiotherapy and Exercise Science, Curtin University, Perth, WA 6102, Australia; angela.jacques@curtin.edu.au (A.J.); kagan.ducker@curtin.edu.au (K.J.D.)

**Keywords:** calcitriol, energy metabolism, muscle strength, lean mass, adults

## Abstract

Supplementation with the most efficient form of Vitamin D (VitD3) results in improvements in energy metabolism, muscle mass and strength in VitD deficient individuals. Whether similar outcomes occur in VitD sufficient individuals’ remains to be elucidated. The aim of this study is to determine the effect of VitD3 supplementation on resting metabolic rate (RMR), body composition and strength in VitD sufficient physically active young adults. Participants completed pre-supplementation testing before being matched for sunlight exposure and randomly allocated in a counterbalanced manner to the VitD3 or placebo group. Following 12 weeks of 50 IU/kg body-mass VitD3 supplementation, participants repeated the pre-supplementation testing. Thirty-one adults completed the study (19 females and 12 males; mean ± standard deviation (SD); age = 26.6 ± 4.9 years; BMI = 24.2 ± 4.1 kg·m^2^). The VitD group increased serum total 25(OH)D by 30 nmol/L while the placebo group decreased total serum concentration by 21 nmol/L, reaching 123 (51) and 53 (42.2) nmol/L, respectively. There were no significant changes in muscle strength or power, resting metabolic rate and body composition over the 12-week period. Physically active young adults that are VitD sufficient have demonstrated that no additional physiological effects of achieving supraphysiological serum total 25(OH)D concentrations after VitD3 supplementation.

## 1. Introduction

Vitamin D (VitD) is an essential pro-steroid hormone [1] responsible for the regulation of calcium and phosphate metabolism [2]. Recently, VitD has received significant scientific interest as the importance of adequate concentration of VitD for optimal skeletal muscle metabolism and function has been identified [3,4]. Interestingly, despite having a temperate climate and many hours of sunshine year-round, one in four Australian adults have a suboptimal VitD status (serum 25(OH)D concentration <50 nmol/L) [2], which highlights the need for further research into the impact of VitD on health and exercise performance. The key source of VitD for humans comes from exposure of the skin to ultraviolet radiation (UVR), allowing the conversion of 7-dehydrocholesterol to cholecalciferol (pre-vitamin D3) and subsequently, to 1,25(OH)2D3 (calcitriol), which is the biologically active form of VitD, representing 80–90% of total VitD production [5].

Vitamin D3 is a potential skeletal muscle modulator and has been reported to influence several muscle functions [6], including skeletal muscle mass and strength, aerobic energy production and lipid metabolism [7]. VitD3 supplementation in healthy adults with low serum concentration of 25(OH)D activates the VitD receptor (VDR) in skeletal muscle, which can stimulate protein synthesis, develop muscle tissue and improve muscle strength in healthy young adults [7,8,9]. Several authors have suggested that supplementation with VitD3 also results in an increase in size and number of type II muscle fibres in VitD deficient individuals [6,10,11]. However, whether these effects can be observed in participants that have adequate or high VitD concentration remains to be elucidated. 

A systematic review by Chiang et al. [12] recently reported that VitD3 supplementation (ranging from 400 to 8500 IU/day) in VitD sufficient athletes resulted in an increase of 1.4–18.8% in muscle strength. The effects of VitD3 supplementation on muscle strength and power has been investigated in soccer [13,14] and rugby players [15], elite ballet dancers [16], swimmers [17] and active adult males [18,19], with mixed findings. However, the majority of these randomized clinical trials (RCT) focused on investigating the effect of VitD in individuals classified as deficient and insufficient at the baseline of the study [13,14,15,16,17,18,19,20]. For example, four RCT [13,16,18,19] reported that VitD3 supplementation increased strength (e.g., isometric force peak, one-repetition maximum (1-RM) bench press, back squat and weighted reverse-grip chin up). Conversely, three RCT reported no effects of VitD3 on any parameter of muscle strength and power [14,17,20]. It is possible that including a population with different concentrations of VitD at the baseline confounds efforts to understand the impact of VitD on muscle strength and power [12].

Currently, the concentration of VitD required for optimal skeletal muscle function is not fully agreed, with guidelines based on the United States Institute of Medicine bone health recommendation of VitD ≥ 50 nmol/L [21,22]. However, it has been suggested by Heaney and Holick [23] that serum total 25(OH)D concentrations of ~120–225 nmol/L may be required for optimal skeletal muscle function, particularly for optimal function. Importantly, long-term oral intake of VitD may cause VitD intoxication (>200 nmol/L) and result in negative health consequences such as gastrointestinal disorders, bone pain, drowsiness and headaches [24], justifying the importance of establishing optimal and safe serum concentration of VitD for skeletal muscle function. 

To date, the majority of intervention studies on the effect of VitD on muscle strength have examined VitD deficient athletes and not those with adequate or supraphysiological concentrations of total VitD. It also seems important to differentiate serum 25(OH)D and the bioavailable free form of VitD (free 25(OH)D), as the latest evidence suggests that VitD binding protein (VDBP) inhibits certain actions of this vitamin, since the bound fraction is unavailable to act on target cells [25,26]. However, it is not clear how the free 25(OH)D versus bound component might impact muscle strength and power in humans [27]. Furthermore, as VitD3 has non-calcemic activities (e.g., inhibition of adipocyte differentiation and remodeling adipose tissue that might impact energy metabolism) [28], it has been suggested that VitD3 may also impact resting metabolic rate (RMR). Identifying variables that affect energy metabolism is essential as changes over time in RMR can have a large impact on fat and muscle mass and vice-versa [29]. Therefore, the aim of this study is to determine the effects of VitD3 supplementation on total and free serum concentrations of 25(OH)VitD, RMR, body composition and strength in VitD sufficient, physically active adults.

## 2. Materials and Methods 

### 2.1. Subjects

A total of 42 physically active adult males and females met the inclusion criteria (exercising at least three times per week with at least two of those sessions involving resistance training; no history of VitD3 supplementation in the last month; no current injuries that would prevent them from completing strength testing; no current use of multivitamins, medication or other supplements that are related with VitD metabolism and body composition (including calcium, thyroxine, creatine and thermogenic supplements)) and were recruited to participate in this study. During the study, four participants had sports injuries unrelated to the study protocol and seven were not able to complete the minimum training required or any of the post-supplementation tests, and then were excluded from this study, leaving a final sample of 31 participants (19 female and 12 male; mean ± SD; age = 26.6 ± 4.9 years; height = 170.0 ± 8.4 cm; body-mass = 71.7 ± 11.8 kg). The study protocol was conducted according to the Declaration of Helsinki and approved by the Curtin University Human Research Ethics Committee (approval number: HRE2019-0028) and registered by the Australian New Zealand Clinical Trials Registry (ACTRN12620000896976). Informed consent was obtained from all subjects.

### 2.2. Study Overview

**Figure 1:**
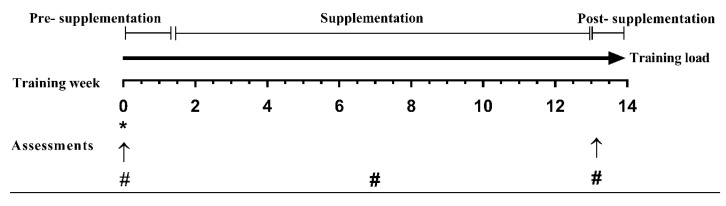
Study design. * Familiarisation with test procedures; ↑ Resting metabolic rate (RMR), body composition and strength and jump tests; # Food intake and venous blood sample.

Participants completed two testing sessions (one pre-supplementation and another post-supplementation) over approximately 13 weeks (Figure 1). First, participants completed initial assessments, including an assessment of RMR, body composition, muscular strength and power and hematological markers [total and free plasma 25(OH)VitD, Ca^2+^ and parathyroid hormone] between week 0 and 1. Data collection started after the summer season (March/April/May, Western Australia) to increase the chance of individuals being VitD sufficient at the baseline and to optimize the chance of participants reaching higher serum total concentration (≥120 nmol/L) at the end of the supplementation period. Following pre-testing, participants were matched for sunlight exposure and randomly allocated in a double-blind and counterbalanced manner to the VitD3 group (total *n* = 17, female *n* = 11; 50 IU/kg body-mass [BM]/day Elite Vitamin D3, Healthspan Ltd.^®^, United Kingdom [UK], batch tested by Informed Sport, LGC Limited, UK) or placebo (total *n* = 14, female *n* = 8; dextrose, Glucodin, iNova Pharmaceuticals, Australia) supplement group for 12 weeks. All doses were concealed in opaque gelatin capsules and organized in sequentially numbered containers to ensure that participants and the testing researcher were blinded to group allocations. 

This dosing strategy was selected because it has been associated with a positive effect on strength performance in previous research [16,18]. In the middle of the study (week 7), hematological markers were again assessed to measure VitD concentration and check for any possible adverse effects of supplementation. Then, following 12 weeks of supplementation, participants repeated the pre-supplementation testing at the same time of day and referred back to their three-day food diary to ensure that they were similarly prepared to perform. Adequate and optimal VitD status were defined as 25(OH)D between 50–100 nmol/L and > 100 nmol/L, respectively, based on previous research that suggests that these concentrations may be related to optimal skeletal muscle outcomes [23,30]. Participants were in contact with the main researcher weekly, to ensure training and supplementation adherence and report any perceived side-effects from the supplementation. Vitamin D3 supplementation was distributed to participants fortnightly and the capsules left were counted to ensure compliance. 

### 2.3. Procedures

#### 2.3.1. Resting Metabolic Rate 

Participants abstained from any strenuous exercise for 24 h prior to the measurement of RMR. Participants arrived at the laboratory as soon as possible after waking following a 12 h overnight fast and were instructed to empty their bladder and void bowels before being weighed. Oxygen consumption was assessed when participants were resting in a supine position, in a quiet room without noise and strong light by metabolic cart (Parvo Medics TrueOne 2400, Parvo Medics, Salt Lake City, UT, USA), using a mouthpiece and mixing chamber. Minute–minute measurements of O_2_ consumption and CO_2_ production were then conducted over a minimum of 30 min. The energy expenditure equation (Weir) was used to calculate RMR from the average of the last 10 min of data collection [31].

#### 2.3.2. Body Composition Assessment

Immediately following the assessment of RMR, participants had their whole-body composition assessed using dual-energy X-ray absorptiometry (DXA; GE Lunar Prodigy, General Electric, Madison, WI, USA), including lean mass, fat mass and total bone mineral density (BMD). Participants completed a pre-scan questionnaire to assess their suitability for scanning, before being positioned on the midline of the scanning bed in a standardized position with both arms by their side with the hands in a mid-prone position within the scan area and feet fixed at a 90° angle at the ankle. All scan analyses were completed using the scanners proprietary software, with adjustments made by the same experienced technician to ensure consistency between scans. 

#### 2.3.3. Assessment of Muscular Strength and Power

Having completed the fasting elements of the testing protocols, the participants were allowed to eat and drink prior to the performance assessment components of the protocol to ensure that they were optimally prepared to perform. First, participants completed a self-selected warm-up, which was recorded and replicated for the post-supplementation testing session. Second, they completed a test of (1-RM) strength for the bench press and back squat exercises following the procedures set out by Baechle and Earle [32]. Briefly, participants lifted progressively greater weights until a mass was identified that could only be lifted for one repetition for that exercise. Finally, participants completed a counter-movement vertical jump test to assess leg power (Vertec Yardstick Jumping Device, Swift, Brisbane, Australia). Participants completed three attempts with a 2–5 min rest between attempts.

#### 2.3.4. Hematological Markers

Participants attended an external pathology laboratory (PathWest Laboratories, Perth, Western Australia) pre-, mid-and post-supplementation, where a venous blood sample (~10 mL) was drawn to assess VitD status, Ca^2+^ and parathyroid hormone. Vitamin D status (25(OH)D) was determined using the Immunoassay method (Abbott Architect i2000sr analyser, Abbott, Illinois, USA). This method has a correlation coefficient (r) of 0.99 with the certified reference method, Isotope Dilution-Liquid Chromatography-Tandem Mass Spectrometry (ID-LC-MS/MS) for 25 (OH)D [33]. Calcium and parathyroid hormone were analysed using the Arsenazo III method and chemiluminescent microparticle immunoassay methodology, respectively (Abbott, Spain). Additionally, two small serum aliquots (1 mL) were separated and frozen to −80 °C for subsequent measurement of the free 25(OH)D concentration. After thawing, concentrations of free 25(OH)D were measured by ELISA immunoassay (DIASource ImmunoAssays, Wijchen, Netherlands) following the procedure described previously [34]. Correlation between rate dialysis analysis results vs. Free 25OH Vitamin D ELISA is described as 0.992.

#### 2.3.5. Training Diary and Questionnaires

As part of a training diary, participants completed a calcium quiz (Dairy Council of California) [35] and sunlight exposure questionnaire [36] on three occasions: baseline, 7 and 12 weeks. Additionally, on these three occasions, participants completed a three-day food record (2 weekdays and 1 day of the weekend), after having received detailed instructions about how to complete their dietary intake. Each food diary was reviewed in detail by a nutritionist together with each participant to ensure that sufficient detail was captured. Food records were analysed using Foodworks^®^ V9 (Xyris Software Pty Ltd., Brisbane, Australia). Each individual was encouraged to follow similar eating patterns throughout the study to minimize deviations in macronutrient, vitamin and mineral intake. Finally, a daily training diary was completed during the 12 weeks of supplementation so that training load could be calculated using the session rating of perceived exertion (sRPE) method proposed by Foster and the adherence to exercise could be confirmed. [37]. Briefly, the participant had to describe the type of exercise, duration of session and chose the rating of perceived exertion for the session (CR1–10 scale) [38]. This value was multiplied by the duration of the session and it provided us with a training load value in arbitrary units (AU).

### 2.4. Statistical Analysis

Categorical data were summarized using frequency distributions. Depending on normality, continuous variables were summarized by means and standard deviations (age, free 25(OH)D, weekly sunlight exposure and daily calcium intake) or medians and interquartile ranges (IQR) [BMI, total 25(OH)D, parathyroid hormone (PTH), Ca^+2^ and total training load). Participants’ main characteristics at pre- and post-supplementation were compared between groups using Chi-squared tests for categorical data and t-tests or non-parametric Mann–Whitney U tests for continuous data. Based on previous research considering strength as the main outcome, a sample size of 30 has 99% power to detect a standardized mean difference of 0.60 (33, 58) in a mixed-model ANOVA in two groups across two time points with an α value of 0.05 [39]. However, due to the ability to adjust for factors, linear mixed modelling was used for the main analyses considering random subject intercepts. Effects of free and total serum 25(OH)D on pre-post supplementation differences in strength, RMR and body composition outcomes, within and between intervention groups, were assessed by linear mixed models (LMM), adjusting for gender, sunlight exposure, training load, protein, carbohydrate, fat and total energy intake. LMM were also used to assess total and serum 25(OH)D concentration within and between groups differences pre- and post-supplementation. This model uses maximum-likelihood estimation methods that parametrize all longitudinal data regardless of missing data points. Results are summarized as estimated marginal means, mean differences and 95% confidence intervals. Statistical significance was as accepted at *p* < 0.05. Analyses were conducted using STATA/IC 16.0 (StataCorp LLC, College Station, TX, USA).

## 3. Results

### 3.1. Participants Characteristics

Participants were equally distributed in both groups in regards to gender, age and ethnicity, showing no significant differences pre-supplementation. The majority of participants were female (*n* = 8 (57%) vs. *n =* 11 (65%); *p* = 0.67) and self-declared Caucasian and white (*n* = 12 (86%) vs. *n* = 15 (88%)) in placebo and VitD3, respectively (*p* = 0.83) (self-reported). On average, participants were 24.9 years ± 4.3 vs. 27.9 years ± 5.3 years old in placebo and VitD3, respectively (*p* = 0.09). Vitamin D3 and placebo group also had similar characteristics including BMI, serum total 25(OH)D concentration, serum parathyroid hormone, serum Ca^2+^, weekly sunlight exposure, sunscreen use, training load, weeks sick or injured and daily calcium intake (Table 1). The VitD3 supplementation dose was calculated by body-mass, which averaged 3205 ± 366 IU/day for females and 4230 ± 548 IU/day for males, respectively. No difference was observed in energy intake (*p* = 0.66), carbohydrate (*p* = 0.06), protein (*p* = 0.77) and total fat consumption (*p* = 0.11) between and within groups during the 12 weeks of supplementation (Abbreviations, Supplementary Figure S1). Additionally, during the supplementation period, participants only forgot to take the VitD3 supplement on average once a month and were sick and/or not training only for 1–2 weeks, confirming an appropriate adherence with supplementation and exercise routine through the full intervention as well as having a very similar training load without any difference between groups (*p* = 0.92).

### 3.2. Serum Total 25(OH)D and Free 25(OH)D Concentration

**Figure 2:**
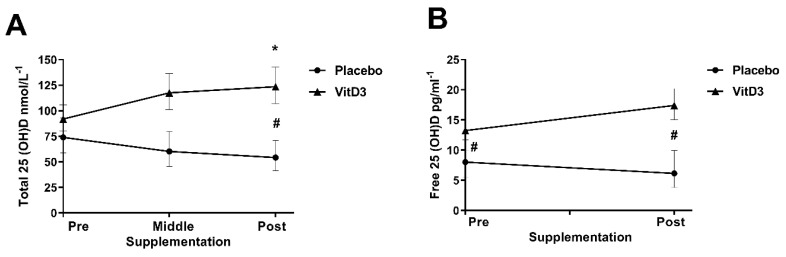
Serum total 25(OH)D concentration pre-, middle- and post-supplementation (**A**) and free 25(OH)D concentration pre- and post-supplementation (**B**). * Difference within group (VitD3 pre-supplementation vs. VitD3 post-supplementation by ANOVA; *p* = 0.01); # difference between groups (VitD3 pre- and post-supplementation vs. placebo pre- and post-supplementation; *p* < 0.001).

Mean baseline serum total 25(OH)D concentration of participants was 87.7 ± 31.4 nmol/L (range = 50.0–175.0 nmol/L), meaning that all participants were classified as VitD sufficient (serum total concentration of ≥50 nmol/L) prior to supplementation (Table 1). Serum free 25(OH)D concentration were higher in VitD3 group when compared with placebo at the baseline of the study (Table 1). Vitamin D3 supplementation significantly increased serum total concentration of 25(OH)D in the VitD3 group (in average by 32%) when compared with baseline (Figure 2A), while the placebo group reduced in average by 28%. A similar effect was not observed when comparing the free 25(OH)D pre-vs. post-supplementation period (Figure 2B). Free 25(OH)D concentration was higher (34%) only at the baseline in the VitD3 group when compared with placebo and this difference was sustained during the study (40%). Three participants from the placebo group had non-detectable serum free 25(OH)D concentrations and were excluded from the analysis.

### 3.3. Muscular Strength and Power

All data from the muscular strength and power tests are reported in Table 2 considering the main predictor as serum total concentration of 25(OH)D and in Table 3 considering the main predictor as serum free 25(OH)D. There were no significant changes between placebo and VitD3 groups in any of the muscle strength and power tests (back squat, bench press and vertical jump displacement) over the 12-week period considering serum total 25(OH)D as the main predictor (1-RM back squat, *p* = 0.54; 1-RM bench press, *p* = 0.38; and vertical jump *p* = 0.50). Within-group increases in strength in the VitD3 group were observed for back squat (+6.9 kg; *p* = 0.005) and bench press (+3.2 kg, *p* = 0.007) (Table 2). Similar within-group increases were observed when free 25(OH)D was considered as the main predictor for back squat (+5.5 kg, *p* = 0.042 vs. +7.7 kg, *p* = 0.003) and bench press (+2.7 kg, *p* = 0.020 vs. +2.9 kg; *p* = 0.01) in both groups (placebo and VitD3, respectively).

### 3.4. Resting Metabolic Rate and Body Composition

Resting metabolic rate, lean mass, fat mass and bone mineral density are described in Table 2 considering serum total concentration of 25(OH)D as the main predictor, and in Table 3 with serum free 25(OH)D as the main predictor. Resting metabolic rate increased in both groups following the 12-week supplementation period (estimated mean difference for placebo = +258.6 kJ and VitD3 = +305.3 kJ); however, there were no significant differences between groups. Within-group increases in RMR in the VitD3 group were observed (RMR + 305.3 kJ, *p* = 0.036 and + 280.9 kJ, *p* = 0.049) considering both predictors (i.e., serum total and free 25(OH)D, respectively). Lean, fat mass and bone mineral density did not differ significantly between the groups after 12 weeks of supplementation with VitD3 or placebo (Table 2). Within-group increases in lean mass (+1.0 kg; *p* = 0.013) in the VitD3 group were also observed when serum total 25(OH)D was the main predictor.

### 3.5. Calcium and Parathyroid Hormone

Serum calcium concentration was constant during the whole study between and within groups (Table 1). Total serum calcium concentration was 2.5 mmol/L (IRQ 0.1) vs. 2.4 mmol/L (IRQ 0.2) pre-supplementation and 2.4 mmol/L (IRQ 0.1) vs. 2.4 mmol/L (IRQ 0.1) post-supplementation in placebo and VitD3 groups, respectively (Table 1), showing no significant differences between placebo and VitD3 groups. In contrast, the concentration of serum parathyroid hormone slightly decreased in the VitD group only, starting with 5.1 (IRQ 3.0) vs. 4.9 (IRQ 3.6) pre-supplementation and with concentration 6.5 (IRQ 2.1) vs. 3.9 (IRQ 3.4) post-supplementation in placebo and VitD3, respectively. Parathyroid hormone concentrations were significantly higher in the placebo group when compared to the VitD3 group (*p* = 0.008; Table 1) post-supplementation. Overall, participants maintained Ca^2+^ and parathyroid hormone serum concentration in the normal range during the study

## 4. Discussion

We describe herein an investigation of the effects of VitD3 on RMR, body composition, strength and power in physically active adults. Following 12 weeks of VitD3 supplementation, total serum concentration of 25(OH)D significantly increased in the VitD3 group; however, no significant differences in RMR, body composition, strength and power were identified when comparing the VitD3 and placebo groups. We also found that supplementation with VitD3 did not significantly change serum free 25(OH)D concentration, which may explain the lack of meaningful effect on the main outcomes.

In our study, the participants demonstrated high concentration of serum total 25(OH)D (placebo = 74 nmol/L; VitD3 = 93 nmol/L) prior to supplementation. These results can be partially explained by approximately 4 h/week of exposure to the intense UVR (mean UVR index = 11–classified as extreme) and high temperatures (mean = 30 °C) during summer months in Perth, Western Australia (situated 31 °S) [40,41]. Vitamin D3 supplementation resulted in a 30 nmol/L (IQR 50) increase in serum total 25(OH)D, while the placebo group reduced total serum concentration by 21 nmol/L (IQR 43) throughout the supplementation period. Therefore, 36% of the placebo group was classified as VitD deficient (<50 nmol/L), with a serum total 25(OH)D concentration of 53 (42.2) nmol/L at the end of this study. Even though the change in serum total 25(OH)D concentration pre- to post-supplementation in the placebo group was not statistically significant, the measured decrease is clinically meaningful as it represents a decrease that may require VitD3 supplementation to maintain adequate concentration throughout the winter months.

Serum total 25(OH)D has a longer half-life than other VitD metabolites and is directly associated with sunlight exposure through dermal synthesis and also dietary intake, justifying why we used it as the primary marker of VitD status [42]. The ‘free-hormone hypothesis’ is considered an alternative pathway for cellular uptake of steroid hormones, as these molecules are highly lipophilic and, therefore, have the potential to quickly and passively diffuse across cell membranes [27]. The initial average range established for the free 25(OH)D concentration is 5.1 pg/mL (2.4–17.1 pg/mL) based on 109 healthy individuals [27]. In the present study, participants had a high concentration of free 25(OH)D (8.6 ± 5.2 pg/mL placebo and 13.1 ± 5.3 pg/mL VitD3 group) before supplementation, which is similar to concentration reported by Sollid et al., 2016 (13.7 ± 4.2 pg/mL) [43]. Surprisingly, free 25(OH)D concentration did not significantly increase after VitD3 supplementation, suggesting participants from the VitD3 group might have already reached optimal serum free 25(OH)D values before starting the intervention protocol.

In our study, participants had an optimal concentration of serum total VitD after supplementation (~120 nmol/L); however, no significant impact on strength or power was detected. This is possibly due to the fact that the majority of participants were already VitD sufficient at the beginning of the study, showing no additional effect of the VitD3 supplementation. Interestingly, within-group differences between VitD and RMR, back squat, bench press, lean mass and bone mineral density, were observed within the VitD3 group [considering serum total 25(OH)D as the main predictor for these outcomes]. In previous RCT studies, the protocols used to measure muscular strength and power were inconsistent, making comparisons with our study challenging [14,16,19,44,45]. Close et al. used a similar protocol to measure strength as we used in our study (1-RM) and they reported that 12 weeks of supplementation with 20,000 or 40,000 IU/week of VitD3 offered no improvements on skeletal muscle strength or power in a VitD deficient, young and active cohort [19]. The authors proposed that higher doses of VitD3 might be needed to reach optimal concentrations of serum total 25(OH)D (>120 nmol/L), as the majority of participants reached only the VitD sufficient range (50–75 nmol/L) previously endorsed for bone health [19].

Clinically, higher dietary VitD intake and serum 25(OH)D concentration is associated with a reduction in omental adipocyte size and lower visceral adiposity in women [46]. Cross-sectional studies report a negative relationship between overweight and/or obesity and serum concentration of 25(OH)D [47], and prospective studies have reported that low 25(OH)D plasma concentration may contribute to the development of obesity [48,49]. In relation to muscle mass, several authors have suggested that supplementation with VitD also results in an increase in size and number of type II muscle fibers in VitD deficient individuals [6,10,11]; however, few RCT have tested these associations in humans directly by muscle biopsy. A recent study that investigated one year of VitD3 supplementation in VitD deficient participants found that lean mass significantly increased from 43.8 ± 9.6 to 44.3 ± 9.8 kg in the VitD group, while no change was observed in the placebo group. In our study, participants maintained a healthy body composition during the 12-week supplementation period and no significant differences were observed after supplementation in lean or fat mass between groups. Randomised clinical trials examining the influence of VitD on energy expenditure are rare. To the best of our knowledge, only one clinical study with a very short supplementation protocol (1 week) reported no influence of VitD3 on energy or substrate utilization and this topic has not been further explored in a physically active population [50]. Cellular studies have reported an increase in control and maximal respiratory capacity, which link oxygen consumption and associated mitochondrial respiration to the generation of ATP, demonstrating a role of VitD in primary human skeletal muscle cell energy production [4,51]. Our results and those of other researchers suggest that the effects of VitD3 supplementation on body composition and RMR might be observed in VitD deficient and/or obese and or/elderly populations, and not in physically active young adults.

It is important for further RCT to perform a dose-escalation study for physically active adults and also to consider the serum free 25(OH)D as part of the total serum 25(OH)D. For example, participants may show different concentration of total versus free 25(OH)D, and therefore, might not be accurately classified as VitD inadequate or adequate (Abbreviations, Supplementary Table S1). Perhaps the recommendation for VitD supplementation in order to correct deficient VitD status should not be based only on total serum 25(OH)D concentration, but also on free 25(OH)D concentrations, and symptoms of VitD deficiency (e.g., fatigue or tiredness, muscle weakness, and bone and muscle pain) [52]. Furthermore, other factors influence the response to VitD supplementation, for example Sollid et al. found that individuals respond better to VitD3 supplementation when they are VitD deficient at the baseline [53].

One limitation of our study is that we were not able to prescribe and control participants’ training. Whilst training loads were similar between group and exercise adherence was high, this may have impacted our findings as we did not observe significant increases in muscle mass in both groups after 12 weeks of resistance training, which would have been expected. Finally, in order to assure that participants would not suffer any adverse effects to supplementation with VitD3 and to guarantee concentration within the recommended range, serum calcium and the PTH were also assessed during the study. Only the concentration of PTH decreased in the VitD3 group in response to the increase of serum total 25(OH)D concentration and the opposite was observed on the placebo group. It is well known that VitD concentration directly impacts serum PTH, helping to regulate calcium metabolism and bone density and function [54]. No adverse effects, including hypercalcemia or hyperparathyroidism were reported for any participant.

## 5. Conclusions

In conclusion, the present study demonstrates no additional benefits on muscle strength, power, RMR and body composition of VitD supplementation in physically active individuals already classified as sufficient in VitD. It is possible that the supplementation of VitD might still be necessary during autumn and winter seasons, where people are at a higher risk to be VitD deficient. Our results suggest that further research using large sample size-controlled trials are required to explore the temporal relationship between serum total and free 25(OH)D concentration with muscle strength and power. We believe that this will assist enhanced understanding of the possible therapeutic effects of VitD supplementation in VitD sufficient and deficient individuals.

## Figures and Tables

**Table 1:** Pre- and post-supplementation characteristics of participants by study group (placebo *n* = 14 and VitD3 *n* = 17).

Parameter	Pre-Supplementation (Week 0–1)		* p-Value	Post-Supplementation (Week 13–14)		# *p*-Value
	Placebo *n* = 14	VitD3 *n* = 17		Placebo *n* = 14	VitD3 *n* = 17	
BMI (kg/m^2^) med (IRQ)	24.8 (3.6)	23.5 (4.6)	0.60	24.7 (4.5)	23.4 (4.5)	0.54
Total 25(OH)D (nmol/L) med (IRQ)	74.0 (44.5) ^a^	93 (49) ^a^	0.07	53 (42.2) ^a^	123 (51) ^b^	< 0.001
Free 25(OH)D (pg/mL) mean (SD)	8.6 (5.2) ^a^	13.1 (5.3) ^b^	0.006	6.2 (4.5) ^a^	15.8 (5.1) ^b^	< 0.001
Parathyroid hormone (pmol/L) med (IRQ)	5.1 (3.0)	4.9 (3.6)	0.68	6.5 (2.1)	3.9 (3.4)	0.008
Ca^+2^ (mmol/L) med (IRQ)	2.5 (0.1)	2.4 (0.2)	0.89	2.4 (0.1)	2.4 (0.1)	0.47
Weekly sunlight exposure mean (SD) (h)	4.4 (4.6)	3.8 (3.1)	0.95	0.7 (0.5)	0.7 (0.5)	1.00
Sunscreen use (yes) *n* (%)	4 (29%)	5 (29%)	0.96	4 (29%)	5 (29%)	0.96
Total training load (AU) med (IRQ)	1635 (1562)	1260 (1065)	0.54	18,840 (16,168)	15,140 (13,995)	0.92
Weeks sick or injured (*n*) mean (SD)	0	0	NA	1.00 (0.5)	1.18 (0.6)	0.42
VitD3 supplementation dose (IU/day) mean (SD)	NA	NA	NA	0	3559 ± 670	NA
Daily calcium intake (mg) mean (SD)	816 (598)	999 (428)	0.09	965 (447)	1109 (482)	0.54

Different letters indicate significant differences between groups pre- and post-supplementation (^a,b^). N/A: not applicable; * *p*-value: comparison between placebo and VitD3 pre-supplementation; # *p*-value comparison between placebo and VitD3 post-supplementation. BMI, total 25(OH)D, PTH, Ca^+2^, total training load are represented by median (IRQ) and Free 25(OH)D, weekly sunlight exposure and daily calcium intake are represented by media ± SD (standard deviation).

**Table 2:** Estimated within-group means and mean differences of outcomes (pre- and post-supplementation) considering serum total 25(OH)D as the main predictor.

Predictor = 25(OH)D		Model Adjusted For: Gender, Sunlight Exposure, Training Load, Protein, Carbohydrate, Fat and Energy Intake									
		Pre-Supplementation			Post-Supplementation			Pre-Post Change			
Outcome	Group	Estimated Mean	95%CI Diff	*p*-Value	Estimated Mean	95%CI Diff	° P-Value	# *p*-Value	Estimated Mean Difference	95%CI	* *p*-Value
Resting Metabolic Rate (kJ)	Placebo	6372.8	5994.6, 6751.1	0.86	6631.5	6221.4, 7041.5			258.6	−48.1, 565.4	0.10
	VitD3	6327.4	6004.5, 6650.4		6632.7	6262.8, 7002.6	1.00	0.83	305.3	20.4, 590.2	0.036
Muscle Strength and Power											
Back Squat (kg)	Placebo	77.9	69.0, 86.9	0.10	82.6	73.2, 92.0	0.07	0.54	4.6	−0.6, 9.9	0.08
	VitD3	87.9	82.6, 94.8		94.8	86.3, 103.4			6.9	2.1, 11.8	0.005
Bench Press (kg)	Placebo	51.3	45.6, 57.1	0.010	52.9	47.0, 58.8	0.005	0.38	1.6	1.1, 4.2	0.24
	VitD3	61.5	56.4, 66.7		64.8	59.4, 70.1			3.2	0.9, 5.6	0.007
Jump test (cm)	Placebo	41.1	36.7, 45.5	0.17	41.9	37.4, 46.5	0.31	0.50	0.8	−0.9, 2.5	0.35
	VitD3	45.2	41.3, 49.2		45.2	41.1, 49.3			−0.01	−1.5, 1.5	0.98
Body Composition											
Lean mass (kg)	Placebo	47.9	47.4	0.09	48.0	44.9, 51.0	0.07	0.54	0.6	−0.3, 1.5	0.19
	VitD3	50.9	50.9		51.9	49.1, 54.6			1.0	0.2, 1.8	0.013
Fat mass (kg)	Placebo	22.7	21.4	0.011	21.4	18.4, 24.5	0.06	0.20	-1.2	−2.5, −0.02	0.050
	VitD3	17.5	17.3		17.3	14.6, 20.1			−0.1	−1.2, 1.0	0.83
Bone Mineral Density (g/cm^2^)	Placebo	1.2	1.7, 1.2		1.2	1.2, 1.2		0.09	−0.0005	0.014, 0.013	0.94
	VitD3	1.3	1.2, 1.3	0.026	1.3	1.2, 1.3	0.008		0.02	0.004, 0.02	0.004

*p*-value: difference between groups pre-supplementation); ° P-value:(difference between groups post-supplementation); # *p*-value: group-time interaction (rate of change); * *p*-value: within group pre-post change. Estimated means and mean differences were assessed by linear mixed models (LMM); 95% CI: confidence intervals; diff: difference.

**Table 3:** Estimated within group means and mean differences of outcomes (pre- and post-supplementation) considering Free total 25(OH)D as the main predictor.

		Model Adjusted For: Gender, Sunlight Exposure, Training Load, Protein, Carbohydrate, Fat and Energy Intake									
		Pre-Supplementation			Post-Supplementation			Pre-Post Change			
Outcome	Group	Estimated Mean	95%CI Diff	*p*-Value	Estimated Mean	95%CI Diff	º P-Value	# *p*-Value	Estimated Mean Difference	95%CI	* *p*-Value
Resting Metabolic Rate (kJ)	Placebo	6361.2	5971.4, 6750.9	0.86	6619.6	6223.8, 7015.5	0.82	0.91	258.4	−39.1, 556.0	0.09
	VitD3	6409.3	6057, 6761.6		6690.1	6296.2, 7084			280.9	0.67, 561.1	0.049
Muscle Strength and Power											
Back Squat (kg)	Placebo	79.1	68.9, 89.4	0.12	84.6	74.2, 95.0	0.08	0.51	5.5	0.2, 10.7	0.042
	VitD3	90.8	80.9, 100.6		98.5	88.0, 11			7.7	2.7, 12.8	0.003
Bench Press (kg)	Placebo	53.3	46.8, 59.7	0.020	56.0	49.5, 62.6	0.030	0.93	2.7	0.4, 5.0	0.020
	VitD3	64.0	57.7, 70.3		66.9	60.3, 73.4			2.9	0.7, 5.0	0.010
Jump test (cm)	Placebo	41.3	36.4, 46.2	0.26	41.6	36.6, 46.6	0.234	0.78	0.3	−1.3, 2.0	0.70
	VitD3	45.4	40.5, 50.2		46.0	41.0, 51.0			0.6	−0.9, 2.2	0.24
Body Composition											
Lean mass (kg)	Placebo	48.0	44.6, 51.3	0.12	48.4	45.0, 51.7	0.10	0.61	0.4	−0.4, 1.2	0.34
	VitD3	51.7	48.4, 54.9		52.3	49.0, 55.7			0.7	−0.1, 1.4	0.09
Fat mass (kg)	Placebo	22.4	19.1, 25.7	0.07	21.6	18.2, 24.9	0.13	0.57	-0.9	−2.2, 0.4	0.19
	VitD3	18.1	14.9, 21.3		17.7	14.4, 21.1			-0.4	−1.6, 0.8	0.55
Bone Mineral Density (g/cm^2^)	Placebo	1.2	1.2, 1.3	0.11	1.2	1.2, 1.3	0.12	0.94	0.007	−0.001, 0.023	0.41
	VitD3	1.3	1.2, 1.3		1.3	1.2, 1.3			0.061	−0.001, 0.021	0.43

*p*-value: difference between groups pre-supplementation); º P-value:(difference between groups post-supplementation); # *p*-value: group-time interaction (rate of change); * *p*-value: within group pre-post change. Estimated means and mean differences were assessed by linear mixed models (LMM); 95% CI: confidence intervals; diff: difference.

## Supplementary Materials

The following are available online at https://www.mdpi.com/2072-6643/12/10/3111/s1, Figure S1: Mean ± (SD) Daily total energy (A) and macronutrient intake (B, C and D) pre-, mid- and post-supplementation in the placebo and VitD3 groups., Table S1: Theoretical method to calculate the percentage of free 25(OH)D availability (example).

## Abbreviations

AU	Arbitrary Units
BMD	Bone mineral Density
IU	International Unit
RCT	Randomized Clinical Trial
RMR	Resting Metabolic Rate
VitD	Vitamin D
VitD3	Vitamin D3
VDBP	Vitamin D Binding Protein
VDR	Vitamin D Receptor
UVR	Ultraviolet Radiation
1-RM	One Repetition Maximum
