# Supplementary material for: Vitamin D Supplementation Does Not Impact Resting Metabolic Rate, Body Composition and Strength in Vitamin D Sufficient Physically Active Adults
Source: Nutrients. 2020 Oct 12;12(10):3111. doi: 10.3390/nu12103111 (PMC7601703; doi:10.3390/nu12103111)
Supplement: Supplementary file 1 [file nutrients-12-03111-s001.pdf]

Supplementary Material

.

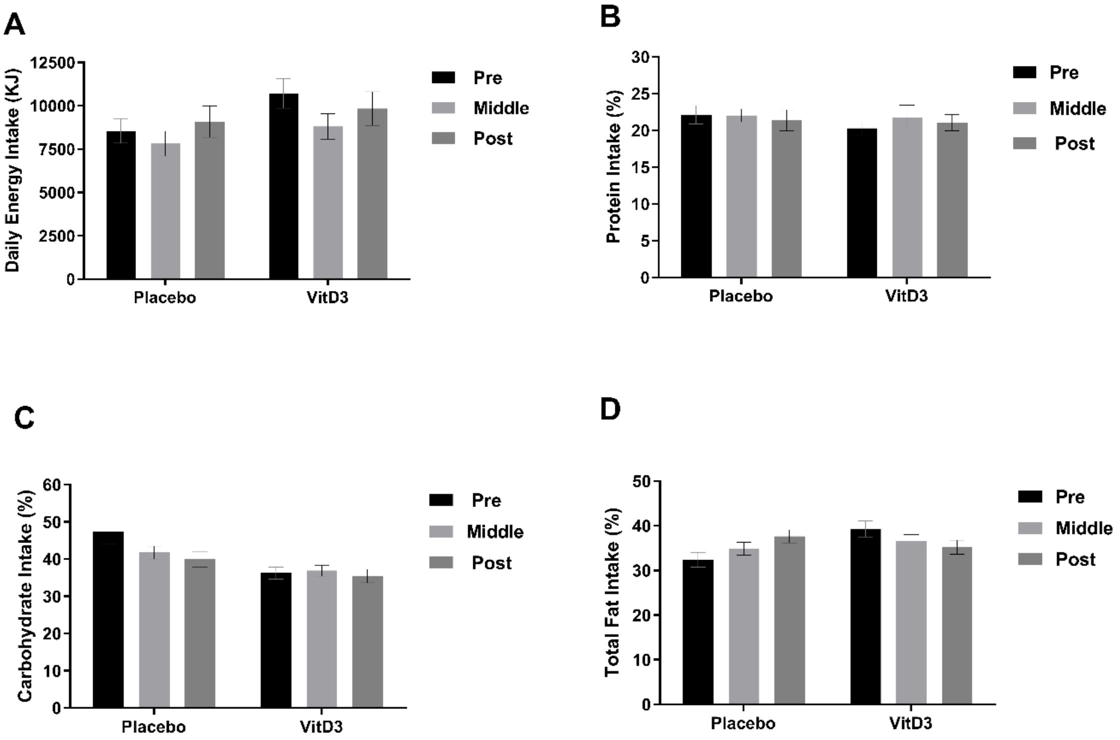

Figure S1. Mean ± (SD) Daily total energy (**A**) and macronutrient intake (**B**, **C** and **D**) pre-, mid- and post-supplementation in the placebo and VitD3 groups.

Table S1. Theoretical method to calculate the percentage of free 25(OH)D availability (example).

Case	Total Serum VitD (nmol/L)	Classification by IOM [21]	Free VitD (pg/ml)	Free VitD (nmol/L)	Free VitD in Proportion to Total VitD	% of Free VitD	Serum Free VitD Fecommendation: 0.03 - 0.4% [53]
1	100	sufficient	2	0.01182	100*0.01182/100	0.01%	Inadequate
2	40	deficient	10	0.0591	100*0.0591/40	0.15%	Adequate
